# A MALDI-TOF nucleic acid mass spectrometry assay for rapid detection of five *Candida* species in urine

**DOI:** 10.3389/fcimb.2025.1682711

**Published:** 2025-11-18

**Authors:** Yi Guo, Yan Chen, Jingfang Sun, Hongxia Zhu, Haiquan Kang, Yinhai Xu

**Affiliations:** 1 Department of Laboratory Medicine, the Second Affiliated Hospital of Xuzhou Medical University, Xuzhou, China; 2 Department of Laboratory Medicine, The General Hospital of Xuzhou Mining Group, Xuzhou, China; 3 Department of Laboratory Medicine, Chongqing General Hospital, Chongqing, China; 4 Department of Laboratory Medicine, Affiliated Hospital of Xuzhou Medical University, Xuzhou, China; 5 School of Medical Technology, Xuzhou Medical University, Xuzhou, China; 6 The Center for Clinical Research and Transformation of Pathogen Diagnosis, Xuzhou Medical University, Xuzhou, China

**Keywords:** *Candida*, urine, MALDI-TOF NAMS, ITS, candiduria

## Abstract

**Background:**

Repeat urine testing is key for the management of candiduria. Current urine testing methods are time-consuming. This study presents an assay coupling PCR with matrix-assisted laser desorption ionization time-of-flight (MALDI-TOF) mass spectrometry (MS), termed MALDI-TOF nucleic acid MS (MALDI-TOF NAMS), for rapid detection of five medically important *Candida* species (*Candida albicans*, *Candida tropicalis*, *Candida glabrata*, *Candida parapsilosis* and *Candida krusei)* in urine.

**Methods:**

Species-specific primers and probes targeting the internal transcribed spacer (ITS) regions the five *Candida* species were designed, followed by PCR amplification, single-base extension, and MALDI-TOF MS analysis. The sensitivity assessment was conducted using *Candida* suspensions and simulated candiduria samples. DNA from bacteria and other fungi was tested to analyse the specificity. To evaluate multiplex detection capability, all five *Candida* species were simultaneously tested in a single-tube reaction. Clinical performance was validated using collected urine samples, with results compared with urine culture.

**Results:**

The developed assay demonstrated a limit of detection (LoD) ranging from 10^1^ CFU/mL to 10^3^ CFU/mL across different *Candida* species, with no cross-reactivity against common uropathogens. The assay allowed simultaneous detection of multiple *Candida* species in a single tube. The diagnostic sensitivity of our developed method was 100% using urine culture as the gold standard, with a diagnostic specificity of 98.7%. The entire detection process could be completed within 5 hours.

**Conclusion:**

The novel MALDI-TOF NAMS assay enables rapid detection of five *Candida* species in urine with validated performance. The assay supports clinical candiduria management and is adaptable for broader *Candida* species detection.

## 1 Introduction

Candiduria is defined as the presence of *Candida* species in the urine. Candiduria is commonly observed among hospitalized patients, particularly those admitted to intensive care units (ICUs) (He et al., 2021b). Additional risk factors include older age, female sex, diabetes mellitus, recent antibiotic use, urinary tract obstruction or instrumentation (e.g., surgery), and indwelling urinary catheterization (Kauffman, 2014; He et al., 2021b). *Candida albicans* was the most common infectious agent causing candiduria, accounting for 50% to 70% of isolates (Alvarez-Lerma et al., 2003; Bougnoux et al., 2008; Kauffman, 2014). Besides, there was a shift towards non-albicans *Candida*, such as *Candida glabrata*, *Candida tropicalis*, *Candida parapsilosis* and *Candida krusei* (Gharaghani et al., 2018; Aghili et al., 2020).

The detection of *Candida* species from urine poses a clinical challenge, as its significance varies from clinically irrelevant contamination or benign colonization to pathogenic urinary tract infections (UTI), each requiring distinct management approaches (Kauffman, 2014; Alfouzan and Dhar, 2017; García-Agudo et al., 2018; He et al., 2021b). As an example, in cases of suspected *Candida* UTI, further species identification is clinically imperative because many isolates of *Candida glabrata* and all isolates of *Candida krusei* are resistant to the first-line antifungal agent (Dias, 2020). Repeat urine testing serves as a critical step to determining the origin of candiduria (Kauffman, 2014; García-Agudo et al., 2018). For instance, an initial repeat clean-catch urine test was suggested to rule out contamination, and a repeat urine test after correcting the predisposing factors (e.g. stopping antibiotics, removing catheter) to assess efficacy (Kauffman, 2014). At present, urine culture is the predominant method used for repeat candiduria testing. However, the process is time-consuming and results are usually not available for several days, making timely management of candiduria difficult.

Molecular biology-based pathogen detection techniques offer potential solutions to this limitation. Polymerase chain reaction (PCR) and its derived techniques, such as quantitative real-time PCR (qPCR), are widely employed for the rapid detection of certain *Candida* from clinical samples (Lima et al., 2017; Walchak et al., 2020; Hernández-Carreón et al., 2021). Matrix-assisted laser desorption/ionization time-of-flight mass spectrometry (MALDI-TOF MS) is another clinically widely adopted technique for pathogen identification, requiring prior culture. The MALDI-TOF nucleic acid MS (MALDI-TOF NAMS) an emerging analytical technique that integrates PCR amplification with MALDI-TOF MS detection. It mainly includes four procedures: (1) locus-specific PCR reaction; (2) shrimp alkaline phosphatase (SAP) treatment to inactivate dNTPs; (3) probe extension reaction; (4) detection of the probe by MS. The probe, an oligonucleotide primer, anneals the sequence in amplified target loci and extends with a single complementary base; the mass signal of the extended probe could be depicted as a peaks at specific position in the spectrum, enabling the culture-independent microorganism identification (Gabriel et al., 2009). Shuai et al. have developed a multiplex MALDI-TOF NAMS method which enables fourteen porcine viruses to be identified at the same time (Shuai et al., 2024). Moreover, the technique has been employed for both species identification within the *Mycobacterium tuberculosis* complex (MTBC) and rapid diagnosis of drug-resistant pulmonary tuberculosis through simultaneous analysis of multiple single nucleotide polymorphisms (SNPs) (Bouakaze et al., 2011; Shi et al., 2025). However, the clinical application of MALDI-TOF NAMS for the detection of fungal pathogens, particularly *Candida* species, has not yet reported in the literature.

Currently, the ribosomal RNA (rRNA) coding genes (rDNA) have been widely recognized as optimal target loci for pathogen detection and identification using PCR-based molecular tools. The rDNA sequences demonstrate significant evolutionary conservation among diverse *Candida* species (Raja et al., 2017). In our previous study, a universal primers/probe system targeting the 5.8S rDNA region was successfully developed, allowing accurate quantification of a wide range of *Candid*a species (Guo et al., 2016). In contrast, the internal transcribed spacer (ITS) regions flanking the rDNA exhibit the higher variation, and are useful for species-level identification (Raja et al., 2017).

Thus, in this study, we developed a MALDI-TOF NAMS assay with specific primers/probe systems targeting ITS regions, allowing the detection of five common *Candida* species in urine samples, including *Candida albicans*, *Candida tropicalis*, *Candida glabrata*, *Candida parapsilosis* and *Candida krusei.* Our method not only provide laboratory basis for the optimal management of candiduria, but also lays the groundwork for the future development of a multiplex detection platform targeting more *Candida* species.

## 2 Materials and methods

### 2.1 Microbial strains and urine specimens

Strains used in this study were classified as reference strains and clinical isolates. Two reference *Candida* species (*Candida albicans* BNCC337321, *Candida tropicalis* BNCC334135) were were purchased from BeNa Culture Collection (BNCC), while the other three (*Candida glabrata* ATCC90030, *Candida parapsilosis* ATCC22019, *Candida krusei* ATCC6258) were obtained from the American Type Culture Collection (ATCC). Clinical strains were recovered from Department of Clinical Microbiology of the Affiliated Hospital of Xuzhou Medical University, including the prevalent pathogens in UTI (*Escherichia coli*, *Enterobacter cloacae*, *Klebsiella pneumoniae*, *Enterococcus faecalis*, *Enterococcus faecium*, *Proteus mirabilis*, *Pseudomonas aeruginosa and Staphylococcus aureus*) and other isolated fungi (*Aspergillus flavus, Aspergillus fumigatus, Cryptococcus neoformans*, *Meyerozyma guilliermondii*, *Trichosporon asahii*, *Candida rugosa*, *Candida metapsilosis*, *Candida orthopsilosis* and *Candida dubliniensis*).

Whole blood and clean-catch midstream urine specimens were collected from healthy volunteers at Xuzhou Medical University’s Health Examination Center, with the urine specimens confirmed pathogen-free by culture for the simulated candiduria study. To evaluate clinical performance, urine specimens were collected from hospitalized patients between June and September 2024. The study protocol was approved by the Institutional Review Board of the Affiliated Hospital of Xuzhou Medical University (Approval No.: XYFY2024-KL178-01).

### 2.2 Sample preparation

Reference strains of *Candida* were inoculated onto Sabouraud dextrose agar (SDA) plates and cultured at 28°C for 48h. The isolated colonies were suspended in sterile double-distilled water, and the concentration was adjusted using an improved Neubauer hemocytometer. Ten-fold serial dilution was performed to obtain suspensions ranging from 10^6^ CFU/mL to 10^0^ CFU/mL for limit of detection (LoD) determination. In addition, 100 μL serial dilutions (10^7^ CFU/mL to 10^1^ CFU/mL) of each *Candida* species were spiked with 900 μL sterile urine from the same healthy volunteer to simulate candiduria (10^6^ CFU/mL to 10^0^ CFU/mL). For specificity assessment, the sterile double-distilled water were spiked with clinical isolates of bacteria and fungi, as described in “1. Microbial strains and urine specimens”.

### 2.3 DNA extraction

The prepared samples or clinical specimens were centrifuged at 12,000 rpm for 5min, and the supernatant was discarded. The pellet was resuspended in 100 μL of nucleic acid extraction buffer (Hybribio, China, Cat. No. HBRT-23) and gently mixed. DNA was then released using a thermal shock method, which involved three cycles of heating at 100°Cfor 5min followed by freezing at -80°Cfor 5min. After centrifugation, the supernatant containing crude DNA extract was collected for downstream analysis. Human genomic DNA was extracted from the whole blood samples according to the manufacturer’s instructions of the commercial kit (TIANGEN, China, Cat. No. DP348-02).

### 2.4 Design of the primers and probes

The rDNA sequences of different strains from the five *Candida* species were downloaded from the Nucleotide database in NCBI, with the requirement that all entries include complete and uninterrupted ITS1 and ITS2 regions. After importing the sequences into DNAMAN for multiple alignment, we retained the conserved regions in ITS1 or ITS2 that showed no variation within species. The selected segments were subsequently loaded into Primer Premier 6 software for the design of primers and probes. This mass-based detection method requires a minimum 30 Da mass difference between between probes and their extension products. In addition, to prevent detection interference from primers, each PCR primer required 5’-terminal incorporation of a 10-nucleotide tag (ACGTTGGATG). The sequences of the primers and probes were given in **Tables 1**, **2**.

**Table 1:** The primers for the MALDI-TOF NAMS assay.

*Candida* species	Locus	PCR primer sequence (5′→3′)
*Candida albicans*	ITS2	F: **ACGTTGGATG**GTTTGGTGTTGAGCAATACGACT
		R: **ACGTTGGATG**CCTAAGCCATTGTCAAAGCGA
*Candida tropicalis*	ITS1	F: **ACGTTGGATG**TTGGTGGCGGGAGCAATC
		R: **ACGTTGGATG**CCGTTGTTGAAAGTTTTGACTATTG
*Candida glabrata*	ITS2	F: **ACGTTGGATG**GCCATATCAGTATGTGGGACACG
		R: **ACGTTGGATG**GTATTAACCCCCGCCGCT
*Candida parapsilosis*	ITS2	F: **ACGTTGGATG**GGGTTTGGTGTTGAGCGATAC
		R: **ACGTTGGATG**GTTTTGGAGTTTGTACCAATGAGTG
*Candida krusei*	ITS2	F: **ACGTTGGATG**GAGCGGACGACGTGTAAAGAG
		R: **ACGTTGGATG**CTCGCAACACTCGCTCTCG

MALDI-TOF: Matrix-assisted laser desorption/ionization-time of flight; NAMS: nucleic acid mass spectrometry; Bold letters indicate the 10-nucleotide tag (ACGTTGGATG).

**Table 2:** The probes for the MALDI-TOF NAMS assay.

*Candida* species	Locus	Probe sequence	Un-extended probe (Da)	Extended base	Extended probe (Da)
*Candida albicans*	ITS2	ACGGTAGTGGTAAGGCG	5315.52	G	5602.72
*Candida tropicalis*	ITS1	ACCGCCAGAGGTTATAAC	5492.61	T	5819.71
*Candida glabrata*	ITS2	CAAACGAGCAGCAGATTAA	5838.89	T	6165.99
*Candida parapsilosis*	ITS2	GTTTGCTTGAAAGAAAGGCG	6221.10	G	6508.30
*Candida krusei*	ITS2	AGCTTCGCTCCCTTT	4469.95	C	4717.15

MALDI-TOF, Matrix-assisted laser desorption/ionization-time of flight; NAMS, nucleic acid mass spectrometry.

### 2.5 PCR reaction

The PCR was performed using the primers described in Section 4 (“Design of the Primers and Probes”) prior to MS analysis. For the PCR reaction, 5 μL purified DNA extract was mixed with 3μL double-distilled water, 1.5μL each of the forward and reverse primers (0.5μM) (Sangon, China), 7 μL reaction buffer and 2μL enzyme (from Zybio MALDI-TOF NAMS preparation kit, China, Cat. No. 01.09.67.01.04.01). The program was run as follows: (1) UNG digestion at 50°C for 2min to prevent carryover contamination; (2) pre-denaturation at 95°C for 5min; (3) 45 cycles of denaturation at 95°C for 10 s, annealing at 58°C for 15 s, and extension at 72°C for 20 s; (4) a final extension at 72°C for 3min. The products were purified using the TIANquick Mini Purification Kit (TIANGEN, China; Cat. No. DP203).

### 2.6 Single-base extension reaction

To dephosphorylate residual dNTPs, 2 µL of shrimp alkaline phosphatase (SAP) and 1µL of reaction buffer (Zybio, China, Cat. No. 01.09.67.01.04.01) were added to the purified PCR products. The mixture was incubated at 37°C for 30 minutes, followed by 85°C for 5 minutes.

For the single-base extension reaction, the products were mixed with 1.5 μL probes (as mentioned in the Section 4), 1.5 μL double-distilled water, 2μL extension buffer and 2 μL extension enzyme (Zybio, China, Cat. No. 01.09.67.01.04.01). After initial denaturation (95°C, 30s), 40 cycles were performed, each comprising: (1) denaturation at 95°C for 5s; (2) 5 iterations of annealing/extension (55°C/80°C, 5s each). Then, the final extension was conducted at 72°C for 3min.

### 2.7 MALDI-TOF MS analysis

**Figure 1:**
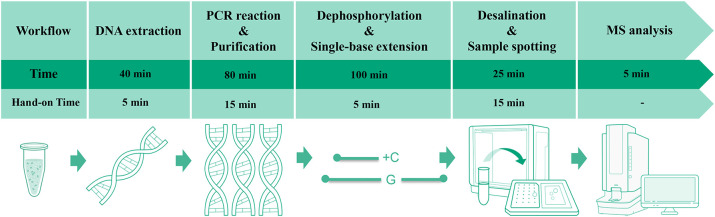
Workflow and time allocation for the MALDI-TOF NAMS assay.

Following the extension reaction, desalting was performed by adding 40 μL double-distilled water and 25 mg disposable resin to each tube. After brief centrifugation, the mixture was vortexed for 10min and then centrifuged. A 20 μL aliquot of the supernatant was spotted onto a matrix-precoated MALDI target plate (Zybio, China, Cat. No. 01.09.67.01.04.01), air-dried, and analyzed by MS (Zybio, China, EXS2600). The complete workflow and time distribution are shown in **Figure 1**.

### 2.8 Method validation

Performance verification of the established method was undertaken as follows: (1) control verification: purified DNA mixtures from five *Candida* species served as positive controls to verify single-base extension products generated by each probe. Sterile water and human genomic DNA were used as blank and negative controls, respectively, to confirm the absence of non-specific extension peaks; (2) sensitivity assessment: each concentration of the serially diluted *Candida* suspensions and simulated candiduria samples was analyzed in 10 replicates. The lowest concentration demonstrating a detection rate >95% and a signal-to-noise ratio (S/N) >3 was defined as the LoD; (3) specificity analysis: genomic DNA from bacteria and other fungi (listed in “1. Microbial strains and urine specimens”) was tested to validate probe specificity; (4) multiplex detection capacity evaluation: DNA extracts from single or five *Candida* species were simultaneously tested against all five probes in a single-tube reaction, and the extension profiles of each probe were observed; (5) clinical validation: urine specimens collected from clinical settings were analyzed using our assay, and the results were compared with those of conventional urine culture.

## 3 Results

### 3.1 The probe peaks in blank and negative controls

**Figure 2:**
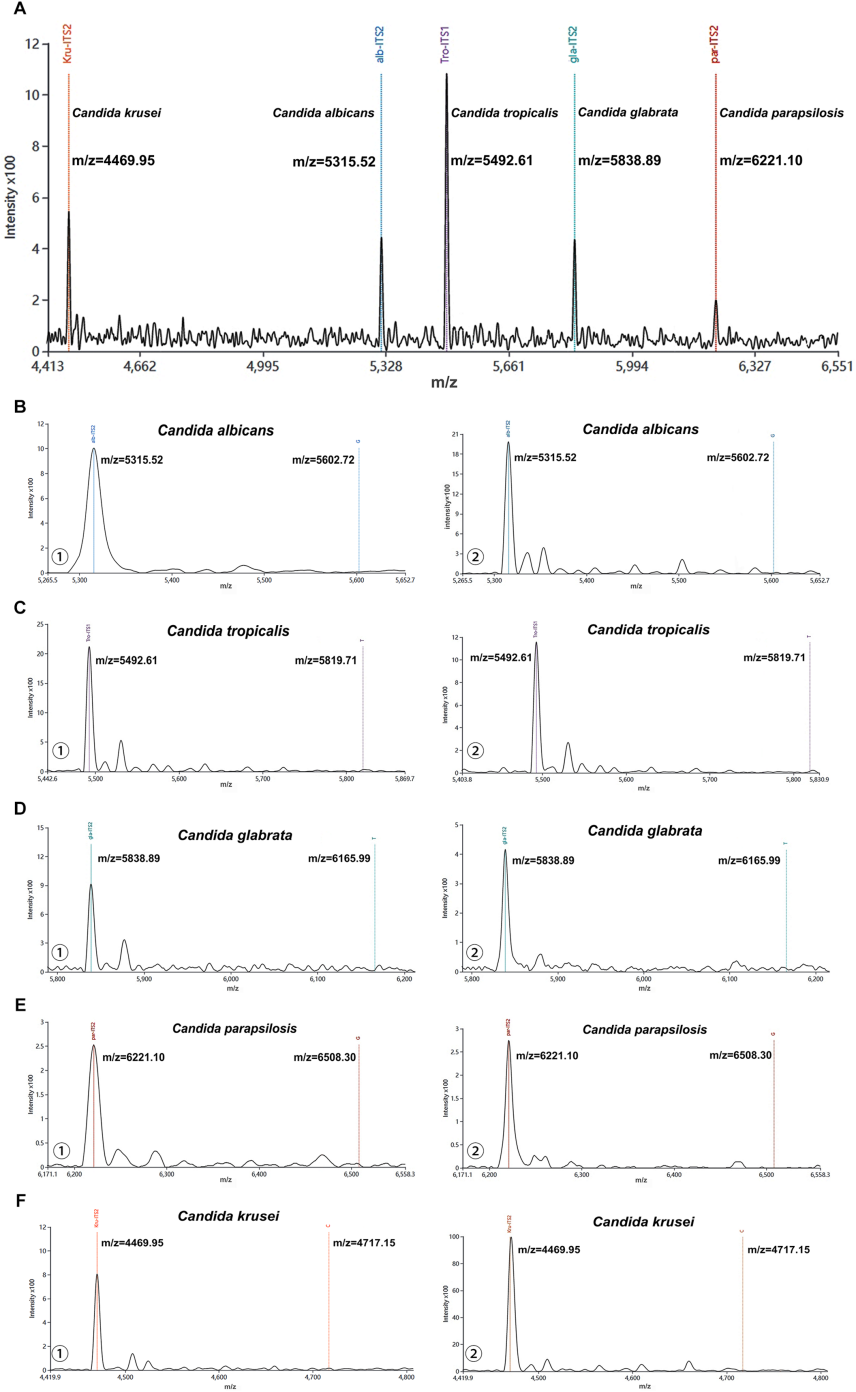
Characterization of probes from five *Candida* species using MALDI-TOF NAMS assay. **(A)** The peak profile of unextended probes from five *Candida* species. **(B-F)** Mass spectra of ① negative and ② blank controls detected by probes from five *Candida* species.

Firstly, the species-specific probes targeting five *Candida* species were directly analyzed by MALDI-TOF MS without prior PCR amplification or single-base extension. **Figure 2A** showed five well-resolved characteristic peaks corresponding to the respective probes at their predicted m/z values, demonstrating excellent mass resolution without any observable signal overlap or cross-interference. Then, the blank control and negative control were analyzed using the MALDI-TOF NAMS protocol. Only a single peak was observed in each mass spectragram of the five *Candida* species (**Figures 2B–F**), indicating that their probes were not extended.

### 3.2 The sensitivity and specificity of the MALDI-TOF NAMS assay

**Figure 3:**
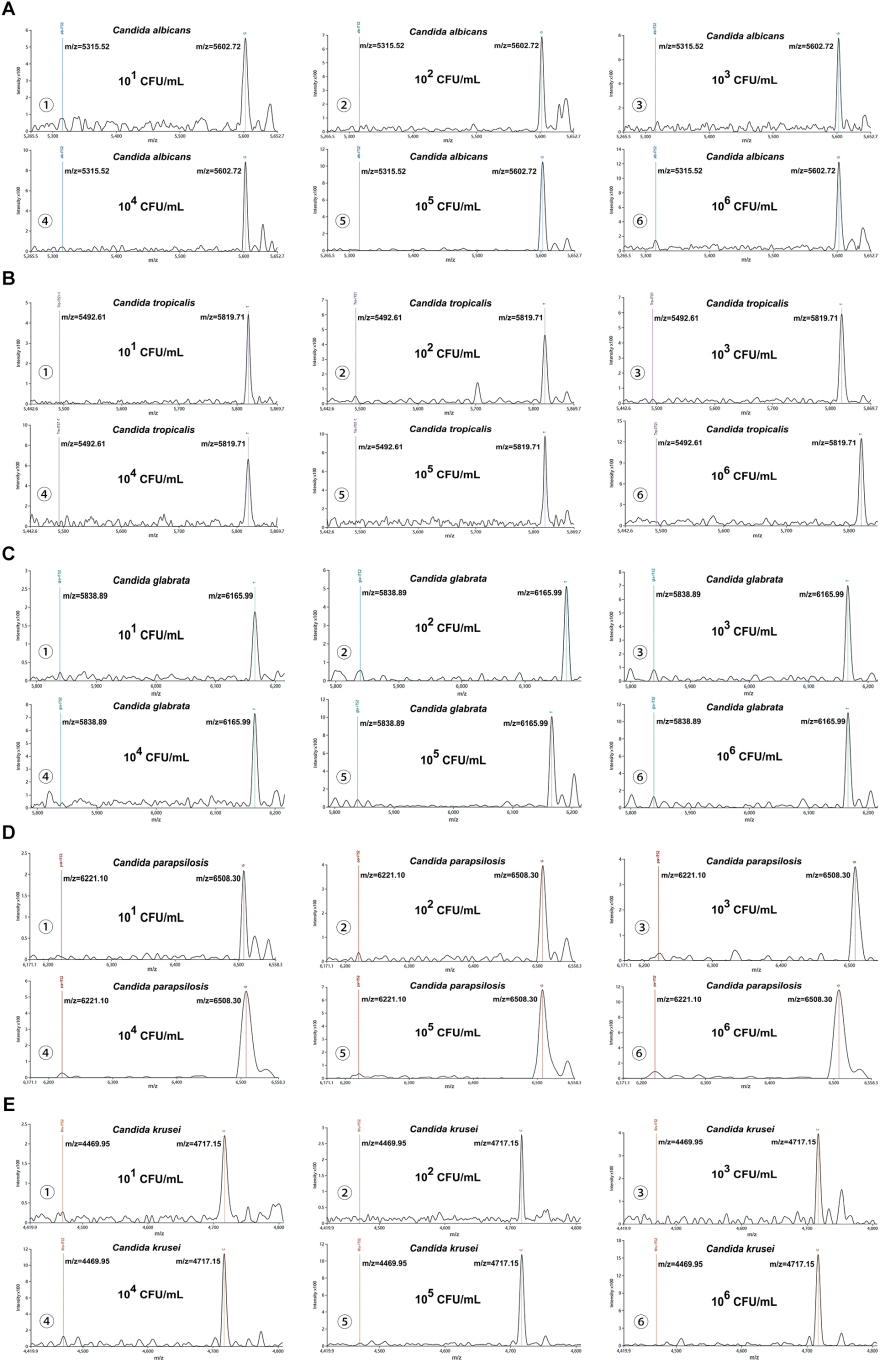
Analytical sensitivity of the MALDI-TOF NAMS assay. **(A–E)** Mass spectra of serial dilutions (10^6^ to 10^1^ CFU/mL) of *Candida* suspensions detected with corresponding species-specific probes.

The MALDI-TOF NAMS detection platform demonstrated 100% detection rates (10/10) for the five *Candida* suspensions at concentrations ranging from 10^1^ CFU/mL to 10^6^ CFU/mL. At 10^0^ CFU/mL, the detection rates varied among species: *Candida albicans* (80%, 8/10), *Candida tropicalis* (60%, 6/10), *Candida glabrata* (50%, 5/10), while *Candida parapsilosis* and *Candida krusei* showed lower detection rates (30%, 3/10 each). Based on a S/N threshold of >3, the LoD for each species were determined as follows: *Candida albicans*, *Candida tropicalis*, and *Candida glabrata* exhibited an LoD of 10^1^ CFU/mL; *Candida parapsilosis* had an LoD of 10^2^ CFU/mL; and *Candida krusei* required a higher concentration (10^3^ CFU/mL) for reliable detection. Details could be seen in **Figures 3A–E**.

**Figure 4:**
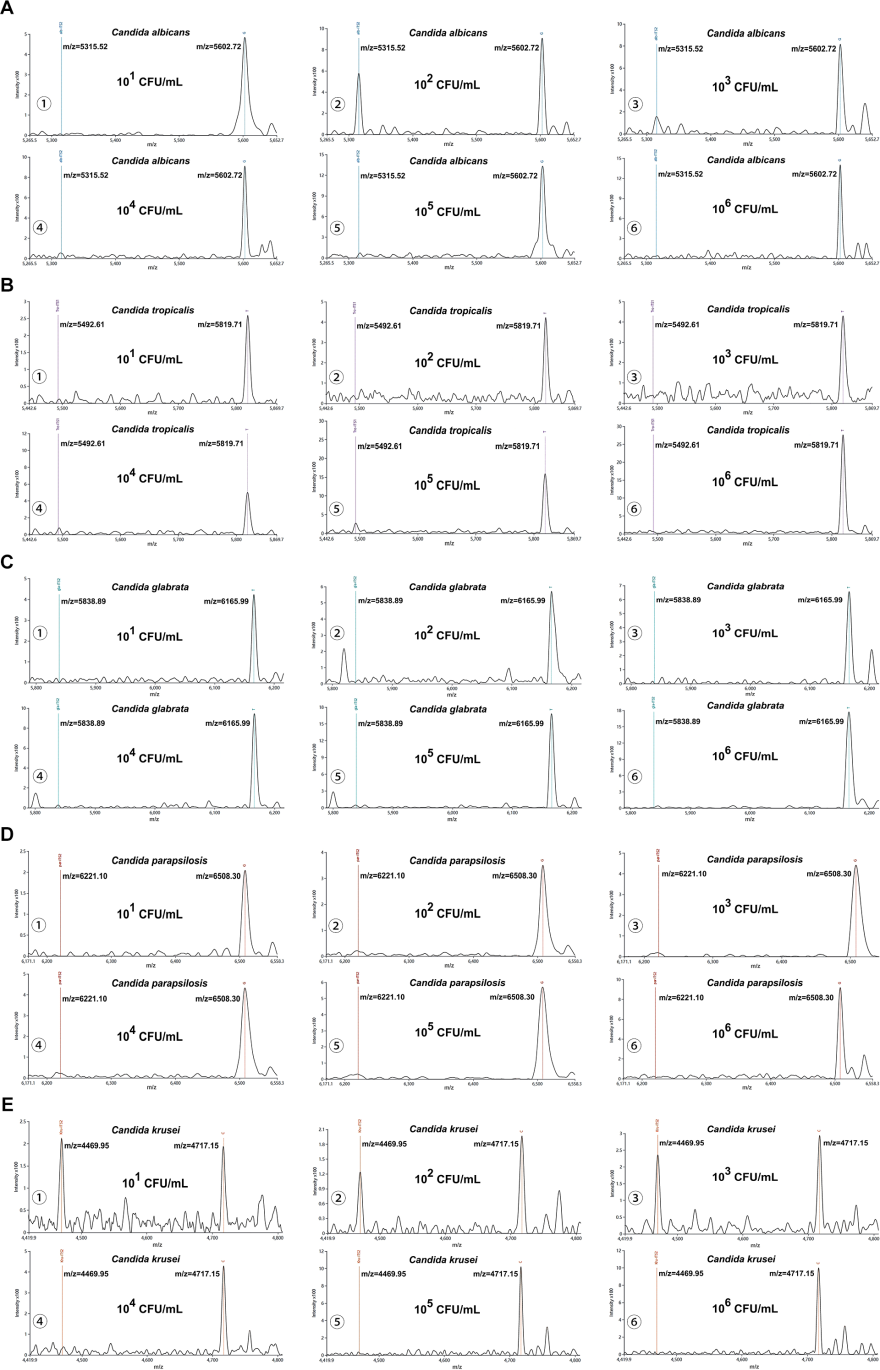
Interference testing of the MALDI-TOF NAMS assay. **(A–E)** Mass spectra of simulated candiduria samples containing *Candida* species at concentrations from from 10^6^ to 10^1^ CFU/mL detected with species-specific probes.

Besides, our platform achieved 100% detection rates (10/10) for simulated candiduria samples across concentrations ranging from 10^1^ to 10^6^ CFU/mL. At the lowest tested concentration (10^0^ CFU/mL), detection rates were observed: *Candida albicans* (70%, 7/10), *Candida tropicalis* and *Candida glabrata* (50%, 5/10 each), *Candida parapsilosis* (40%, 4/10), and *Candida krusei* (30%, 3/10). Using a S/N threshold of >3 as the criterion for reliable detection, the LoD for simulated candiduria were established as follows: 10^1^ CFU/mL for *Candida albicans*, 10^2^ CFU/mL for *Candida tropicalis*, *Candida glabrata*, and *Candida parapsilosis*, and 10^3^ CFU/mL for *Candida krusei* (**Figures 4A–E**).

To evaluate the specificity of our methodology, genomic DNA from eight bacterial strains and nine non-target fungal species was individually analyzed as templates. Results indicated the presence of only non-extended probe peaks, confirming the high specificity of our detection system for the five targeted *Candida* species without any cross-reactivity with other non-target pathogens.

### 3.3 The multiplex MALDI-TOF NAMS assay

**Figure 5:**
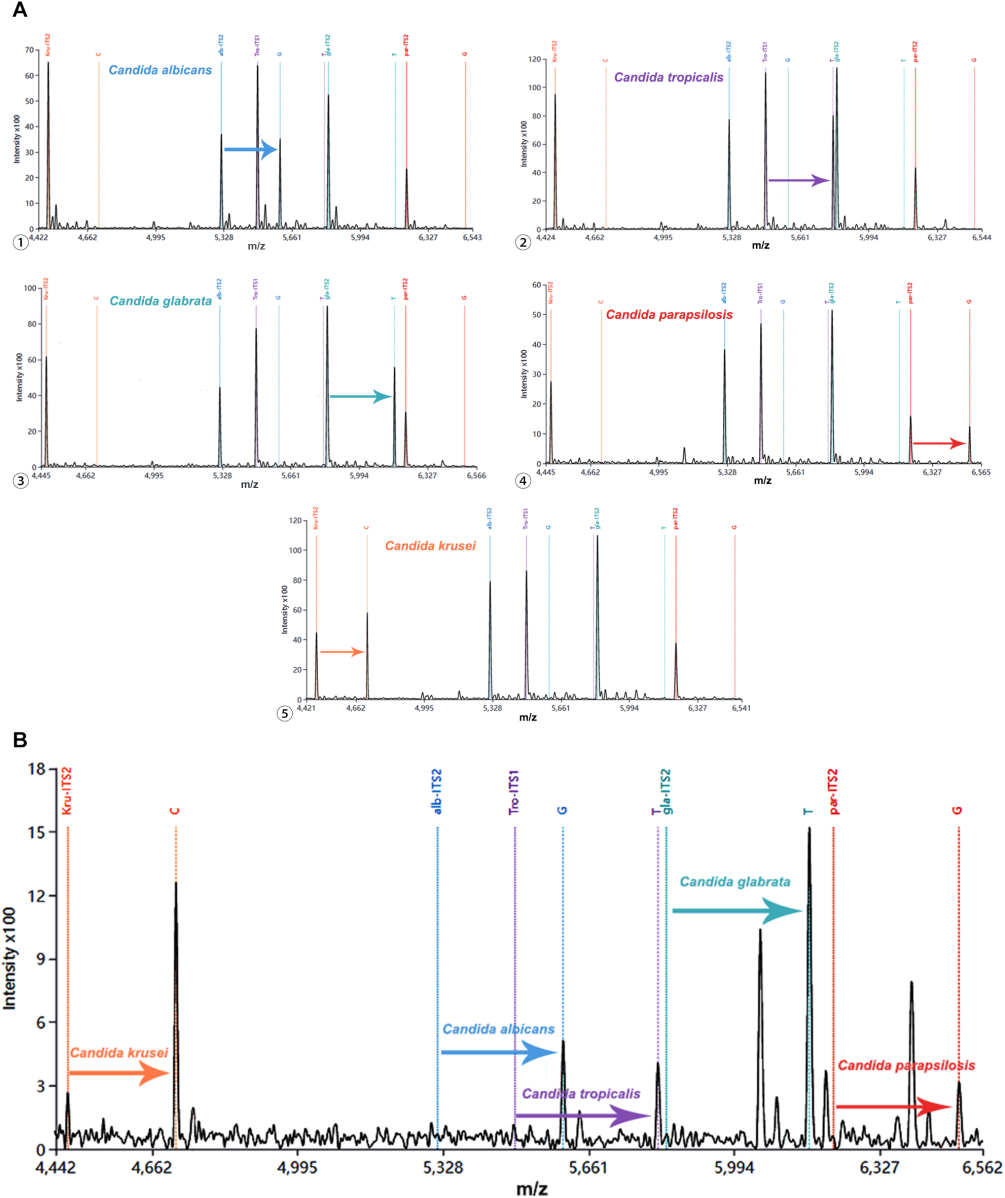
Multiple detection of five *Candida* species using MALDI-TOF NAMS assay. **(A)** Individual detection of each species: ① *Candida albicans*, ② *Candida tropicalis*, ③ *Candida glabrata*, ④ *Candida parapsilosis* and ⑤ *Candida krusei*; **(B)** Simultaneous detection of all five species in a mixed sample. MALDI-TOF, Matrix-assisted laser desorption/ionization-time of flight; NAMS, nucleic acid mass spectrometry.

To evaluate the multiplex detecition capacity of our detection system, we employed a multiplex assay in which the amplified products derived from certain *Candida* species were hybridized with five probes in a single tube. As illustrated in **Figure 3A**, the probe specific for *Candida albicans* successfully extended, generating a characteristic peak that was clearly distinguishable from those non-extended probes. Similarly, probes for remaining four *Candida* species demonstrated selective single nucleotide extension, generating their identifiable peaks respectively (**Figure 5A**). Moreover, when the amplicons from five *Candida* strains were mixed with all species-specific probes in a single reaction, the characteristic peaks corresponding to each extended probe were unambiguously identifiable (**Figure 5B**). These results demonstrate that the developed detection system enables the simultaneous identification of at least five clinically relevant *Candida* species, providing a robust basis for candiduria analysis in the clinical settings.

### 3.4 Performance evaluation of MALDI-TOF NAMS using clinical urine samples

A total of 1,844 urine samples from patients were test using urine culture and our developed method. Urine culture showed that 31 specimens were positive for *Candida albicans*, 20 for *Candida tropicalis*, 9 for *Candida glabrata*, 1 for *Candida parapsilosis* and 1 for *Candida krusei*, with the mean (standard deviation, SD) turnaround time of 2812.3 (1012.6) minuets.

The 62 urine specimens with *Candida* were obtained from 41 patients with a mean (SD) age of 70.6 (18.8) years. Among them, 61.0% (n=25) were female. The majority of the patients were admitted to the ICU (36.6%, n=15) and internal medicine departments (53.7%, n=22), while the remaining patients were distributed in the surgery-urology department (7.3%, n=3) and burn unit (2.4%, n=1). The past histories of the patients were prevalent, with diabetes mellitus (70.7%), renal diseases (29.2%) and urinary tract obstruction (21.9%) being the most common. Additionally, 33 (80.5%) patients had other chronic conditions, particularly hypertension, coronary heart disease and cerebral infarction. Most patients underwent various procedures. Urinary catheterization was performed in 82.9% (34/41) of cases, antibiotic therapy was administered to 90.2% (37/41) of patients, while nasogastric tube insertion and abdominal surgery were required in 53.7% (22/41) and 14.6% (6/41) of cases, respectively.

Almost half of the patients (n=19, 46.3%) failed to receive any form of clinical management after the presence of candiduria. Urine culture was only repeated in 31.7% (n=13) of the patients with candiduria. Except for that, bladder irrigation represented the most frequently performed medical intervention (n=9, 22.0%), followed by catheter replacement (n=6, 14.6%) and antibiotic discontinuation (n=3, 7.3%). Only one patient (2.4%) had his urinary catheter removed. Eight patient (19.5%) were finally diagnosed as *Candida* UTI and administered antifungal therapy. Moreover, 2 patient (4.9%) progressed to candidemia. The relevant data were presented in **Table 3**.

**Table 3:** Demographics and clinical characteristics of 41 patients with candiduria.

Variable	Overall (n = 41)
Age (years)	70.6±18.8
Female sex	25 (61.0%)
Onset at ICUs	15 (36.6%)
Underlying morbidity	
Diabetes mellitus	29 (70.7%)
Renal diseases	12 (29.2%)
Urinary tract obstruction	9 (21.9%)
Other chronic diseases	33 (80.5%)
Procedures	
Urinary indwelling catheters	34 (82.9%)
Antibiotic use	37 (90.2%)
Nasogastric tube usage	22 (53.7%)
Abdominal surgery	6 (14.6%)
Numbers of testing	
Once	28 (68.3%)
Twice or more	13 (31.7%)
Diagnosis	
*Candida* UTIs	8 (19.5%)
Candidemia	2 (4.9%)

ICU, the intensive care unit; UTI, urinary tract infections.

Our species-specific probes successfully identified all *Candida* to the species level in the 62 candiduria samples. Besides, extension peaks were also detected in 23 urine specimens that were culture-negative for *Candida*, of which 10 corresponded to *Candida albicans*, 12 to *Candida tropicalis*, and 1 to *Candida glabrata.* Consequently, the diagnostic sensitivity our developed method was 100.0% when using urine culture as the gold standard, and the diagnostic specificity was 98.7%. **Table 4** provides a comprehensive summary of the clinical evaluation data.

**Table 4:** Comparison of MALDI-TOF NAMS and urine culture results using clinical urine specimens.

	Urine culture		Total
MALDI-TOF NAMS	+	-	
+	62	23	85
–	0	1759	1759
Total	62	1782	1844

## 4 Discussion

In the present study, we established a novel multiplex MALDI-TOF NAMS assay for the simultaneous identification of five prevalent *Candida* species associated with candiduria. The developed assay demonstrated a LoD ranging from 10^1^ CFU/mL to 10^3^ CFU/mL across different *Candida* species, with no observed cross-reactivity against a panel of UTI pathogens and commonly encountered fungi. The diagnostic sensitivity of our developed method was 100% using urine culture as the gold standard, with the diagnostic specificity of 98.7%. The entire detection process could be completed within 5 hours, since our assay circumvented the need for conventional incubation procedures of urine specimens.

The prevalence of candiduria differs across patient populations. Colodner et al. reported that the occurrence of community-acquired candiduria was 0.14%, while the prevalence of candiduria from outpatients and inpatients was 0.77% (Colodner et al., 2008). Among catheterized hospitalized patients, the incidence of candiduria rose to 19.49% (Padawer et al., 2015). In ICU settings, the proportion of patients with candiduria reached was as high as 22.00% (Alvarez-Lerma et al., 2003). Our study population encompassed general ward patients, catheterized patients, and ICU patients, placing our findings within the range of results reported in previous studies (62/1844, 3.36%). In a regional healthcare complex comprising three hostipals providing tertiary general care services, Castellano-Sánchez et al. reported that the isolation rate of *Candida* spp. from urine culture was 2.72% (Castellano-Sánchez et al., 2025), which was similar to our result.

Candiduria often receives insufficient clinical consideration, leading to neglected management in routine practice. Most patients with candiduria receive no medical intervention with a range of 36.3% to 81.4% (Colodner et al., 2008; Padawer et al., 2015; He et al., 2021a), and our finding (46.3%) also falls within this range. According to the Infectious Diseases Society of America (IDSA) guidelines, at least one clean-catch repeat urine culture is required to rule out contamination, even when candiduria is detected in an asymptomatic non-catheterized patient (Pappas et al., 2016). In high-risk candiduria cases, follow-up urine testing should be performed after addressing the underlying predisposing conditions (Kauffman, 2014). It is the urine culture’s laborious process that somewhat impacts clinical decisions on candiduria management. In our study cohort, five patients were discharged on the day following candiduria detection, precluding clinicians from awaiting repeat culture results and consequently resulting in no medical intervention.

PCR-based molecular methods enable rapid detection of candiduria and identification of specific *Candida* species. Hernández-Carreón et al. introduced a fast endpoint PCR method to reliably identify *Candida glabrata* in urine specimens (Hernández-Carreón et al., 2021). To rapidly and directly detect *Candida auris* from urine samples, Walchak et al. developed a real-time PCR assay utilizing specific primers and probes (Walchak et al., 2020). Few common isolates in candiduria could be identified through the PCR-RFLP analysis with *MspI* restriction enzyme digestion (Ortiz et al., 2018; Fazeli et al., 2019). However, conventional PCR methods were limited in the number of pathogens they could simultaneously detect within a single panel (Zhang et al., 2021). In addition, their insufficient resolution hindered the accurate differentiation of closely related species, such as *Candida albicans* and *Candida dubliniensis* (Ortiz et al., 2018).

Compared with other traditional PCR-based assays, our method employed MS to analyze amplified DNA fragments, thereby achieving high resolution and multiplex detection capability. The assay discriminates closely related *Candida* species (e.g., *Candida albicans* vs. *Candida dubliniensis*; *Candida parapsilosis* vs. *Candida orthopsilosis*/*metapsilosis*) down to single-base differences, while allowing simultaneous detection of multiple *Candida* species in a single tube. Moreover, unlike conventional MALDI-TOF MS for proteins, our MS assay targeted PCR products, enabling culture-independent identification of five clinically relevant *Candida* species directly in urine within 5 hours (**Figure 1**).

The developed platform demonstrated high sensitivity (10^1^ CFU/mL to 10^3^ CFU/mL), meeting the clinical diagnostic requirements for UTI. When combining recombinase polymerase amplification (RPA) with lateral flow strips (LFS) for the detection of *Candida glabrata* and *Candida tropicalis*, the sensitivity was limited to 10^4^ CFU/mL and 9940 CFU/mL, respectively (Wang et al., 2022; Wang et al., 2022). Zhao et al. reported a lower LoD of 10 CFU/50 µL (200 CFU/mL) for *Candida krusei* using the same technology; however, this finding lacked validation through replicate testing (Zhao et al., 2022). Of note, the current method showed higher sensitivity for suspensions than for simulated candiduria samples when detecting *Candida glabrata*, *Candida tropicalis*, and *Candida parapsilosis*, which may be attributed to matrix effects inherent in clinical specimens (Munch et al., 2019). There are some advantages of our methodology that we believe differentiate it from commercial kits (**Table 5**). Firstly, commercial kits may not always be readily accessible in certain regions, particularly in resource-limited settings. In contrast, our in-house designed panel is more adaptable to local needs, easier to produce, and more cost-effective. Moreover, our methodology offers the flexibility to tailor the panel to clinical requirements, focusing on *Candida* species that are most prevalent in the local population, such as *Candida albicans*, *Candida glabrata* and *Candida tropicalis*. This adaptability enhances the clinical utility of our method, particularly in settings where these species are the primary concern.

**Table 5:** A head-to-head comparison of the reported molecular methods in the literature for the candiduria detection.

Method	Target gene	Sensitivity (suspensions)	Sensitivity (simulated samples)	Clinical validation	Standard	Cost	Turnaroud time	*Candida* spp.	Reference
Endpoint PCR	^1^NE	3.3×10^3^-3.3×10^4^ CFU/mL	3.3×10^3^ - 3.3×10^4^ CFU/mL(blood)	Y	Not described	Low	Not described	*Candida glabrata*	Hernández-Carreón et al., 2021
Real-time PCR	^2^ITS	^3^1.85×10^2^ CFU/mL	^4^3.75×10^2^ CFU/mL (urine)	N	–	Moderate	^5^One day	*Candida auris*	Walchak et al., 2020
^6^RPA-LFS	^2^ITS	^7^10^4^ CFU/mL	Not described	Y	Real-time PCR	Low	Within 30 min	*Candida glabrata*	Wang et al., 2022
^6^RPA-LFS	^2^ITS	^8^9.94×10^3^ CFU/mL	Not described	Y	Real-time PCR	Low	Within 30 min	*Candida tropicalis*	Wang et al., 2022
^6^RPA-LFS	^2^ITS	^9^2×10^2^ CFU/mL	Not described	Y	Real-time PCR	Low	Within 30 min	*Candida krusei*	Zhao et al., 2022
^10^MALDI-TOF NAMS	^2^ITS	10^1^-10^3^ CFU/mL	10^1^-10^3^ CFU/mL(urine)	Y	Urine culture	Moderate	Within 5 hours	*Candida albicans* *Candida glabrata* *Candida tropicalis Candida parapsilosis Candida krusei*	This study

^1^: NE: *cis*-acting regulatory element localized downstream from *EPA1*; ^2^: ITS: internal transcribed spacer; ^3^: 37 CFU/200μL reported in the literature; ^4^: 75 CFU/200μL reported in the literature; ^5^: On the day of patient admission; ^6^:RPA-LFS: recombinase polymerase amplification combined with lateral flow strip; ^7^:10 CFU/µL reported in the literature; ^8^: 9.94 CFU/µL reported in the literature; ^9^:10 CFU/50 µL reported in the literature; ^10^:MALDI-TOF NAMS: matrix-assisted laser desorption ionization time-of-flight nucleic acid mass spectrometry.

In clinical validation, our assay successfully identified *Candida* to species level in all culture-positive urine specimens. 23 NAMS positive results were obtained in 1782 culture-negative specimens. The diagnostic specificity aligns with established literature reports, ranging from 93.0% to 99.0% (Eshoo et al., 2010; Bacconi et al., 2014; Desmet et al., 2016). Among the 23 culture-negative but NAMS-positive specimens, more than half (12) were from patients who had received antifungal therapy. The antifungal treatment may have eradicated the *Candida* cells, leading to negative urine cultures (Facchini et al., 2024), but the DNA of the non-viable *Candida* remained in the urine, resulting in positive PCR-based detection. In 4 culture-negative/NAMS-positive cases, subsequent urine cultures from the same patients later grew *Candida*, matching the species identified by MALDI-TOF NAMS. This may be related to the lower sensitivity of culture methods (Clancy and Nguyen, 2013). As the disease progresses, *Candida* load in the urinary tract increases, resulting in positive cultures in follow-up samples. The remaining patients most likely represented temporary *Candida* presence in the urine (Wellinghausen et al., 2009). The low *Candida* load may have been cleared by the host immune system, making urine cultures negative, but the released DNA can still be detected by PCR-based methods. Nevertheless, the possibility of a false-positive PCR result cannot be completely ruled out (Wellinghausen et al., 2009). *Candida* is a common commensal organism in the human body, particularly colonizing the oral cavity, gastrointestinal tract, and skin (Romo and Kumamoto, 2020). During specimen collection, *Candida* from the skin may contaminate the urine container. While low-level *Candida* loads may not yield positive cultures due to insufficient microbial growth or viability, highly sensitive PCR assays could still amplify trace DNA derived from contaminants.

Besides, it should be noted that the method established in this study presents several other limitations. Firstly, The current assay mainly focuses on the five most prevalent *Candida* species in the clinical setting. However, the MALDI-TOF NAMS platform is inherently scalable and can be readily expanded to include additional probes for emerging or clinically relevant *Candida* species, such as *Candida auris*, *Candida dubliniensis* and *Candida lusitaniae*. The detection principle of MALDI-TOF NAMS is based on mass differences among nucleic acid extension products (Gabriel et al., 2009). Therefore, in theory, there is no fundamental limitation on the number of targets that can be detected simultaneously, provided that the probes are well-designed to avoid mass overlap and non-specific interactions. For instance, a recent study successfully applied MALDI-TOF NAMS to simultaneously detect 14 porcine viruses in a single assay, demonstrating the feasibility of high-plex detection in complex samples (Shuai et al., 2024). This supports the adaptability of our platform for future inclusion of additional *Candida* species as needed. Secondly, while the current assay is specifically validated for urine samples, the underlying MALDI-TOF NAMS holds considerable potential for adaptation to other clinical specimens, such as blood or respiratory samples. Preliminary studies on various clinical samples are needed to assess the assay’s performance, as different specimen matrices may impact detection. Additionally, *Candida auris*, which has emerged as a significant global pathogen with multidrug resistance, is not currently included in the assay, but could be considered for future inclusion for surveillance purposes. Expanding the assay’s scope would enhance its utility in broader clinical settings. Finally, the performance of the multiplex assay remains to be systematically further validated.

## 5 Conclusion

In this study, we developed a novel MALDI-TOF NAMS assay for the rapid identification of five clinically prevalent *Candida* species (*Candida albicans*, *Candida tropicalis*, *Candida glabrata*, *Candida parapsilosis*, and *Candida krusei*) in urine samples. The assay demonstrated superior sensitivity, excellent specificity, and high concordance rate with urine culture results. Its multiplex detection capability and streamlined workflow address critical limitations of conventional methods, offering a robust tool to differentiate contamination, colonization or UTI for timely candiduria management in high-risk populations.

## Data Availability

The original contributions presented in the study are included in the article, further inquiries can be directed to the corresponding author.
